# A Case–Control Study of the Effects of *Chimarrão* (*Ilex paraguariensis*) and Coffee on Parkinson's Disease

**DOI:** 10.3389/fneur.2021.619535

**Published:** 2021-03-10

**Authors:** Márcio Schneider Medeiros, Artur Francisco Schumacher-Schuh, Vivian Altmann, Carlos Roberto de Mello Rieder

**Affiliations:** ^1^Serviço de Neurologia, Hospital de Clínicas de Porto Alegre, Porto Alegre, Brazil; ^2^Departamento de Farmacologia, Universidade Federal do Rio Grande do Sul, Porto Alegre, Brazil; ^3^Departamento de Neurologia, Universidade Federal de Ciências da Saúde de Porto Alegre, Porto Alegre, Brazil

**Keywords:** *Ilex paraguariensis*, Parkinson's disease, coffee, caffeine, risk factor

## Abstract

**Introduction:** Coffee has been inversely associated with Parkinson's disease (PD) in many studies, and caffeine is the leading candidate to mediate this effect. *Mate* (*Ilex paraguariensis*, IP), a caffeinated beverage rich in antioxidants consumed in South America, was also inversely associated with PD in one study from Argentina. Other varieties of IP infusion, such as *chimarrão*, were never studied in PD. *Chimarrão* is a common caffeinated beverage consumed in Brazil made with the leaves and stems of IP.

**Methods:** A case–control study was conducted to evaluate the relationship between *chimarrão* ingestion and PD in southern Brazil. All subjects answered a questionnaire about the frequency of *chimarrão* and coffee intake. A multiple regression analysis adjusted for age and sex was performed to assess the association between PD and *chimarrão* consumption.

**Results:** We included 200 PD patients and 200 healthy controls. High consumption of *chimarrão* was inversely associated with PD (OR = 0.44, 95% CI = 0.24–0.81, *P* = 0.008). High consumption of coffee was also inversely associated with PD, as expected. *Chimarrão* remained associated when adjusted for coffee consumption, smoking history, and age (OR 0.46, 95% CI = 0.25–0.86, *P* = 0.014). These two exposures showed an additive effect.

**Conclusion:** *Chimarrão* consumption was inversely associated with PD, even after adjusting for coffee intake, suggesting a possible protective role. IP's effect can be mediated by caffeine and through its antioxidant components. *Chimarrão* has a lower concentration of caffeine compared with coffee and has numerous substances with antioxidative effects that may be important to PD protection. Further studies are needed to test this hypothesis.

## Introduction

The etiology of Parkinson's disease (PD) is not fully understood, and different environmental factors have been associated with PD. These factors are thought to either enhance the risk of developing the disease or provide a protective effect. Coffee is inversely associated with PD, and this effect seems to be mediated by caffeine (1). Several studies have found this association, supporting the evidence of the protective effect of caffeine in PD (1, 2), with a more evident outcome in men (2). Other caffeinated beverages have also been studied, especially tea (3), corroborating the protective role hypothesis of caffeine on PD. Caffeine acts on adenosine A2 receptors on dopaminergic D2 neurons in the *substantia nigra*, a mechanism potentially implicated with a neuroprotective effect (4). By antagonizing these receptors, caffeine alters dopamine transmission, protects against glutamatergic excitotoxicity, and frees radicals such as nitric oxide (5).

*Chimarrão*, a hot infusion of *Ilex paraguariensis* (IP), is a common caffeinated beverage consumed in Brazil. IP is a native plant from South America, and *chimarrão* was first consumed by its indigenous people. In the Seventeenth century, it was adopted by Spanish and Portuguese colonizers, and nowadays, it is consumed daily by ~30% of the population in southern Brazil (6). It is also popular in Uruguay, Argentina, and Paraguay, where it is called *mate*. However, *chimarrão* contains both leaves and stems, different from the *mate* consumed in Argentina, which is usually free of stems. *Mate* was previously associated with lower PD risk in Argentina, and caffeine was considered the probable mediator of this effect (7). Previous studies have demonstrated a high content of antioxidants and substances with iron chelation properties in IP, which could elicit a potential protective effect on PD (8, 9). In a recent study by Bernardi et al. (10), they demonstrated that IP may have a strong neuroprotective activity on dopaminergic neurons, preventing their death with a dose-dependent effect (10).

The hypothesis of a neuroprotective effect of IP infusions on PD is understudied. Whether this effect is due to caffeine alone or in combination with antioxidants is yet to be demonstrated. We conducted a case–control study to further investigate the effect of IP on PD.

## Methods

This is an observational analytic (case–control study) based on a single cohort of 233 Parkinson's disease subjects from the Movement Disorders Clinic at a University hospital in Porto Alegre, Brazil. All patients were selected between 2006 and 2012 and evaluated by neurologists with training in movement disorders (AFSS and MSM), and the diagnosis of PD was defined according to the UK Parkinson's Disease Society Brain Bank Research criteria (11) and followed until 2019. This cohort has been described elsewhere (12). A subset of 200 patients answered surveys with information about coffee and *chimarrão* intake, smoking history, and disease characteristics, such as age at onset and disease duration in the years 2016–2017. Inclusion criteria were patients with a diagnosis of PD and no evidence of cognitive decline defined by the Mini Mental State Examination. We excluded patients younger than 45 years of age and individuals diagnosed with vascular or atypical parkinsonism. From a study group at the same hospital, 200 healthy individuals without any neurological conditions, including cognitive decline, were selected as the control group in the same period, between 2016 and 2017. Control subjects were excluded if there was any suspicion of a neurodegenerative disorder and the possibility of drug-induced parkinsonism. Patient and control groups were matched for sex and age. Sample size was calculated using the following parameters: a matched case–control study, an expected odds ratio of 0.64, the prevalence of *chimarrão* consumption in southern Brazil of 30%, an alpha level of 0.05, a power of 0.90, and a moderate correlation between case and control exposures for matched pairs (0.2). The estimated sample size was 116 cases and 116 controls.

Study subjects from both case and control groups answered an environmental exposure questionnaire where the frequency and quantity of *chimarrão* and coffee consumption were estimated. The number of specific cups used for *chimarrão* (*cuias*) and coffee was recorded in the following categories: never, < 2 cups/*cuias* per week, 2–6 cups/*cuias* per week, 1–2 cups/*cuias* per day, 3–5 cups/*cuias* per day, and 6 or more cups/*cuias* per day. Data on years of consumption for both beverages were also collected to estimate lifelong intake. Smoking history was categorized as ever vs. never, and a history of smoking at least 100 cigarettes during a lifetime was required to be considered as a positive smoking history.

*Chimarrão* has a lower concentration of caffeine (8–27 mg/150 ml) compared with coffee (58–109 mg/150 ml) (13), and to control for this difference, two variables representing low and high levels of consumption of each caffeinated beverage were created. In these variables, high consumption of *chimarrão* (daily consumption of at least 6 *cuias*) and high consumption of coffee (daily consumption of at least 3 cups) were defined.

Two final variables of total caffeine intake were created. First, a variable was created to identify the difference between individuals who never consumed caffeinated beverages and individuals who consumed both beverages. The second variable evaluated the amount of caffeine when both beverages were consumed: no caffeinated beverages, low consumption of both *chimarrão* and coffee, and high consumption of both *chimarrão* and coffee.

This study was approved by the local ethics committee and all subjects provided written informed consent.

### Statistical Analyses

All statistics were performed with R 3.5.2. Data on age and years of ingestion of *chimarrão* and coffee were compared between the two groups with Student *t*-test. Differences in sex and individuals with positive smoking history were analyzed using χ^2^ test. Logistic regression analysis was performed to assess the effect of *chimarrão* and coffee intake on PD (using the variables for consumption defined above), adjusting for possible confounding variables.

## Results

We included 200 PD patients and 200 controls. There was no statistical difference concerning sex and age. Both groups had ~50% male individuals. The mean age for cases and controls were 65.69 (SD = 10.52) and 67.35 (SD = 9.23), respectively. The mean disease duration for PD patients was 8.78 (SD = 6.05) years. There was no difference in years of *chimarrão* and coffee consumption between the groups. Smoking history was significantly higher in the control group (*P* < 0.001). Sociodemographic characteristics of the population and consumption of *chimarrão* and coffee are shown in Table 1.

**Table 1:** Demographic data of cases and control subjects.

	**Cases**,	**Controls**,	***P*-value**
	***n* = 200**	***n* = 200**	
Male, %	50.5	49.5	0.920
Age, mean (SD)	65.69 (10.52)	67.35 (9.23)	0.093
Age at onset, mean (SD)	57.16 (11.29)	–	–
Disease duration, mean (SD)	8.78 (6.05)	–	–
***Chimarrão*** **ingestion (*****cuias*****), %**			
- Never	49.0	40.5	
- Less than 2 per week	11.5	5.5	
− 2–6 per week	7.5	11.0	
− 1–2 per day	9.0	3.5	
− 3–5 per day	12.0	15.0	
− 6 or more per day	11.0	24.5	
**Coffee ingestion (cups), %**			
- Never	21.5	8.0	
- Less than 2 per week	10.0	2.5	
− 2–6 per week	11.5	8.0	
− 1–2 per day	44.0	60.5	
− 3–5 per day	13.0	19.0	
− 6 or more per day	0.0	2.0	
**Years of consumption, mean (SD)**			
- *Chimarrão*	43.47 (18.33)	44.55 (16.16)	0.645
- Coffee	54.86 (15.92)	55.96 (14.62)	0.513
Smoking history, count	58	102	<0.001

Univariate analysis showed a significant association of high consumption of *chimarrão* with PD (OR 0.37, 95% CI = 0.21–0.66, *P* = 0.001) but no association of low consumption (*P* = 0.798). As for coffee, low and high ingestions were associated with PD (OR 0.31, 95% CI = 0.19–0.50, *P* < 0.001 and OR 0.27, 95% CI = 0.14–0.50, *P* < 0.001, respectively). Smoking history was also inversely associated with PD (OR 0.39, 95% CI = 0.26–0.59, *P* < 0.001). There was no association of the duration of *chimarrão* and coffee consumption and PD diagnosis. Age was not significantly associated as well (Table 2).

**Table 2:** Univariate analysis of *chimarrão* and coffee consumption, smoking history, years of consumption, and age (*chimarrão* low consumption <6 *cuias*/day; *chimarrão* high consumption ≥6 *cuias*/day; coffee low consumption <3 cups/day; coffee high consumption ≥3 cups/day).

**Variables**	***n***	**OR**	**95% CI**	***P*-value**
***Chimarrão***				
- Low	150	0.94	0.61–1.46	0.798
- High	71	0.37	0.21–0.66	0.001
**Coffee**				
- Low	209	0.31	0.19–0.50	<0.001
- High	68	0.27	0.14–0.50	<0.001
Smoking history	160	0.39	0.26–0.59	<0.001
**Years of consumption**				
- *Chimarrão*	216	1.00	0.98–1.01	0.643
- Coffee	335	1.00	0.98–1.01	0.512
Age	400	0.98	0.96–1.00	0.094

In the logistic regression analysis, after adjusting for smoking history and age, the high consumption of *chimarrão* remained associated with PD (OR 0.44, 95% CI = 0.24–0.81, *P* = 0.008). Low and high consumption of coffee also remained associated with PD (Table 3).

**Table 3:** Model of *chimarrão* and coffee consumption adjusted for smoking history and age (*chimarrão* low consumption <6 *cuias*/day; *chimarrão* high consumption ≥6 *cuias*/day; coffee low consumption <3 cups/day; coffee high consumption ≥3 cups/day).

**Variables**	***n***	**OR**	**95% CI**	***P*-value**
***Chimarrão***				
- No consumption		Ref		
- Low	150	1.01	0.64–1.58	0.981
- High	71	0.44	0.24–0.81	0.008
**Coffee**				
- No consumption		Ref		
- Low	209	0.34	0.21–0.55	<0.001
- High	68	0.28	0.15–0.52	<0.001
Chimarrão^*^				
- No consumption		Ref		
- Low	150	0.97	0.61–1.54	0.891
- High	71	0.46	0.25–0.86	0.014

* *Adjusted for coffee consumption*.

The *chimarrão* model was adjusted for coffee consumption and the high consumption category remained inversely associated with PD independent of smoking history and age (OR 0.46, 95% CI = 0.25–0.86, *P* = 0.014).

Finally, a total caffeine effect was assessed by the variables including the intake of both *chimarrão* and coffee. The first analysis identified a lower OR when both beverages are consumed compared with either beverage alone (OR 0.23, 95% CI = 0.10–0.55, *P* = 0.001). The second variable (divided in three categories based on the quantity of caffeine consumed—no consumption, low consumption of both *chimarrão* and coffee, and high consumption of both *chimarrão* and coffee) indicated a strong association of low and high total caffeine consumption and PD, again with lower ORs compared to the either beverage alone (OR 0.28, 95% CI = 0.12–0.66, *P* = 0.003 and OR 0.20, 95% CI = 0.09–0.48, *P* < 0.001, respectively) (Table 4).

**Table 4:** Evaluation of total caffeine as no consumption and consumption of both beverages and total caffeine as both beverages in low vs. high consumption—multivariate analysis adjusted for smoking history and age.

**Variables**	***n***	**OR**	**95% CI**	***P*-value**
**Total caffeine**				
- No consumption		Ref		
- Both beverages	200	0.23	0.10–0.55	0.001
**Total caffeine**				
- No consumption		Ref		
- Low quantity	199	0.28	0.12–0.66	0.003
- High quantity^*^	163	0.20	0.09–0.48	<0.001

* *High quantity = either both chimarrão and coffee in high quantity or at least one in high quantity and the other in low quantity*.

## Discussion

The present study shows a lower consumption of *chimarrão* among PD patients compared with matched controls, suggesting that it can be a protective factor for the disease. In Argentina, *mate* was associated with lower rates of PD in one previous study (7), and other caffeinated teas were also inversely associated with PD in different countries (14, 15). Studies with Japanese and Chinese populations showed protective effects of black tea independent of coffee intake (3).

With regard to the consumption of *chimarrão*, individuals in the control group showed a higher intake compared with PD patients. In Argentina, *mate* consumption was also inversely associated with PD in that population (7). Interestingly, Gatto et al. described a dose-dependent effect of *mate* with ORs ranging from 0.50 to 0.23 for intakes of 0.5–1 L/day and more than 1 L/day, respectively (7). The fact that in our study only high *chimarrão* consumption showed an inverse association with PD could be explained by the lower concentration of leaves in *chimarrão* compared with the Argentinian *mate*. This could lead to different concentrations of caffeine in the two kinds of IP infusion. Therefore, if both coffee and *chimarrão* have protective effects due to their caffeine content and its effect on adenosine A2 receptors, lower concentrations of caffeine in *chimarrão* [about one third compared with a cup of coffee (13)] may explain why only the high consumption group was associated with lower PD.

*Chimarrão* is widely consumed in southern Brazil. Although it is similar to the *mate* prepared in other South American countries, the Brazilian infusion usually contains more stems than the Argentinian *mate*, made only with the leaves. The leaves are where the studied compounds of the plant are found, including not only caffeine, but also other substances with antioxidative activity. The Brazilian blend contains more powder than other blends, has a vibrant and intense green color, and is readily packed after the harvest, unlike the Argentinian type which is left to age.

Among the substances found in IP, several can mediate a possible neuroprotective effect. One of its important compounds, chlorogenic acid (CGA), attenuated lipid and protein oxidation in a model of chronic immobilization stress (CIS) in rats (8). It also prevented the decrease in superoxide dismutase (Sod) and catalase (Cat) activities after induced seizures in the brains of Wistar rats (9). In another study with oxidative stress by De Lima et al. CGA was unable to prevent all the alterations induced by CIS, suggesting that the protective properties of IP are the result of the combined effects of all its antioxidant compounds, which includes caffeine (16). Bixby et al. in 2005 found a potent, dose-dependent protective effect against oxidative stress and the highest inhibition of protein nitration in IP compared with wine and green tea (17). The fact that the total caffeine variables were highly associated with lower PD individuals and had lower OR compared with the separate consumption of the two drinks indicates that both beverages have additive effects. Although no definite conclusions can be taken from that, this could suggest that IP and coffee may not have the same mechanisms of action—IP may act through caffeine and antioxidants.

More recently, Bernardi et al. (10) showed that IP has protective effects on dopaminergic neurons, preventing neuronal death in cell culture (10). Our study did not evaluate IP composition and the concentration of caffeine and antioxidants, but both mechanisms should be considered. *Chimarrão* may be less concentrated than other IP infusions, but it is possible that even more concentrated versions, as the *mate* consumed in Argentina, may not reach the concentration of antioxidants tested in animal and cellular studies.

In our sample, coffee consumption was lower among PD patients, and it corroborates the idea that coffee confers protection for PD, being inversely associated with PD in several studies (1, 2, 18). Caffeine also presents a possible symptomatic effect as well as an interference in disease progression. It has been associated with reduced accrual of motor and non-motor symptoms in a 4-year follow-up study in Italy and corroborated by a recent meta-analysis (19, 20). In addition, it has also been inversely associated with the development of dyskinesias (21).

Smoking is usually related to PD as a protective factor, frequently associated with coffee intake (22, 23). In our study, the results of the caffeinated drinks remained inversely associated after adjusting for smoking history.

### Study Limitations and Strengths

The self-reported data, the estimation of ingestion, and caffeine concentration used in this study are limitations to be considered because of a memory bias. Patients had to remember how many cups they generally drank per week and for how many years. This bias is due to our study design, but it should be considered that the estimation was used in both case and control groups, limiting a possible negative effect of this bias. Also, we should consider as limitations the fact that we were unable to define the size of cups and *cuias* used by each individual, and it was not possible to evaluate the concentration of caffeine in the coffee and *chimarrão* consumed.

Finally, we should highlight that our study included patients from a movement disorder clinic increasing diagnostic accuracy, controls were matched for age and sex, and smoking history was included in the analysis.

## Conclusion

*Chimarrão* was inversely associated with PD in the category of high consumption individuals. This is the first time *chimarrão* is studied in the context of Parkinson's disease, bringing new information concerning IP and neurodegeneration. Coffee also showed a possible protective effect on the disease as different studies have previously shown. These two exposures exhibited an additive effect, and it is not possible to conclude that *chimarrão* acts only through its caffeine content. New evidence of neuroprotection points to possible effects of other antioxidant substances in *I. paraguariensis*, although our results cannot support this hypothesis. Designing studies with a more accurate ingestion record, measuring the levels of caffeine/antioxidants consumed, and controlling dietetic sources of caffeine pose methodological challenges, but an effort in this direction would be warranted to confirm these findings.

## Data Availability Statement

The raw data supporting the conclusions of this article will be made available by the authors, without undue reservation.

## Ethics Statement

The studies involving human participants were reviewed and approved by Comitê de Ética do Hospital de Clínicas de Porto Alegre. The patients/participants provided their written informed consent to participate in this study.

## Author Contributions

MM contributed with data collection, analysis, and manuscript elaboration. VA participated in data collection and manuscript elaboration. AS-S and CR helped with the study concept and were important to interpret the results and to revise the final manuscript. All authors contributed to the article and approved the submitted version.

## Conflict of Interest

The authors declare that the research was conducted in the absence of any commercial or financial relationships that could be construed as a potential conflict of interest.
